# Pathways from physical exercise to life satisfaction among Chinese university students: The chain mediating role of exercise adherence and health literacy

**DOI:** 10.3389/fpubh.2026.1721049

**Published:** 2026-04-09

**Authors:** Zhong-lei Cui, Wen-hao Zhang, Wei Wang, Shan-shan Han, Sai Zhu, Chuan-yi Xu, Hua-sheng Huang, Rong-hai Luo

**Affiliations:** 1 Physical Education College of Shangqiu Normal University, Shangqiu, China; 2 Institute of Sports Science, Nantong University, Nantong, China; 3 Nantong Health College of Jiangsu Province, Nantong, China; 4 Guangxi University of Chinese Medicine, Nanning, China

**Keywords:** chain mediation, college students, exercise adherence, health literacy, life satisfaction, physical exercise

## Abstract

**Objective:**

This study aimed to investigate the influence of physical exercise on life satisfaction among college students. To further explore the underlying mechanisms, two mediating variables were introduced: exercise adherence and health literacy. This approach helped analyze the pathway through which physical exercise predicts and affects students’ life satisfaction.

**Methods:**

A total of 12,573 valid questionnaire responses were collected from 13,042 college students in East China, as listed in the 2024 Ministry of Education announcement of ordinary colleges and universities. The established scales included in the questionnaire are the Physical Activity Rating Scale (PARS-3), the Satisfaction with Life Scale (SWLS), the Exercise Adherence Scale, and the Health Literacy Scale (HLS-SF9). The data were analyzed using the chain mediation model and the bias-corrected Bootstrap method.

**Results:**

Most college students (73.1%) engaged in a low level of physical exercise. Significant positive correlations were found among physical exercise, health literacy, exercise adherence, and life satisfaction (r = 0.137–0.954). After controlling for demographic variables, physical exercise negatively predicted life satisfaction directly (*β* = −0.010, *p* < 0.001). It also influenced life satisfaction through three indirect pathways. The separate mediating effect of exercise adherence was significant (effect = 0.040, 95%CI[0.036,0.043]). The chain mediating effect of exercise adherence and health literacy was also significant (effect = 0.014, 95%CI[0.012,0.015]). The separate mediating effect of health literacy was not significant. These results suggest that exercise adherence and health literacy play a partial chain mediating role between physical exercise and life satisfaction.

**Conclusion:**

According to the measurements from the Satisfaction with Life Scale, physical exercise has a significant negative correlation with life satisfaction among college students. Results show that physical exercise can positively influence the life satisfaction of Chinese university students through the chain mediating effect of exercise adherence and health literacy. Future research, using larger and more diverse samples, should further investigate the effects of factors such as age, types of exercise, and duration of activity.

## 1 Introduction

Positive Psychology is a significant branch of psychology. It has developed into an academic field that systematically studies core issues such as positive psychological traits, subjective well-being, and life satisfaction. As a key dimension, life satisfaction serves as a core indicator for assessing individual mental health. It is also an important parameter for evaluating overall social development (1). It is noteworthy that the college student population is in a critical transitional phase from “student” to “social member.” This particular period is often accompanied by relatively high mental health risks (2). Exploring the mechanism between physical exercise and the life satisfaction of college students has thus become a significant research topic.

Life satisfaction refers to an individual’s subjective evaluation of their quality of life based on their own standards. It is often regarded as a comprehensive indicator of happiness (3). Michalski Cad’s study found that lower life satisfaction may significantly increase an individual’s likelihood of using mental health services in the future (4). Separately, the Central People’s Government of China has issued important guidance. Along with the Ministry of Education and other relevant agencies, it emphasizes the importance of prioritizing student mental health. This policy is part of the ‘Action Plan for Comprehensively Strengthening and Improving Student Mental Health Work in the New Era (2023–2025)’. The plan encourages explicitly physical exercise to improve mental well-being. It further advocates sports activities as practical tools for emotion regulation and stress relief (5). Thus, greater attention should be paid to the mental health of university students. More importantly, it is essential to identify factors that influence psychological well-being and provide support for enhancing life satisfaction.

Physical exercise, as a modifiable behavioral factor, has a sustained positive effect on life satisfaction (6). Physical exercise is a comprehensive form of physical activity aimed at promoting physical and mental health (7). Physical exercise not only enhances the physiological functioning of college students but also serves as an effective intervention for improving life satisfaction. Selye’s endorphin hypothesis suggests that physical exercise effectively stimulates the release of neurotransmitters such as endorphins. This process modulates the levels of excitatory neurotransmitters, thereby alleviating stress experiences. Ultimately, this leads to enhanced life satisfaction (8). A longitudinal study by Guang-yu examined Chinese college students. The results showed that life satisfaction scores increased significantly with higher exercise intensity (9). Diane K. Ehlers conducted a study on older adults. Her research confirmed that individuals with strong autonomous motivation sustain well-being through regular exercise. This, in turn, indirectly improves life satisfaction (10). A report from the U. S. Centers for Disease Control and Prevention indicates that regular exercise 3–5 times per week significantly improves psychological status. This effect is particularly pronounced among individuals with low life satisfaction (11). Existing research has confirmed a significant association between physical exercise and life satisfaction. However, the underlying psychological mechanisms remain to be further elucidated. Revealing these mechanisms will provide a scientific basis for developing more effective exercise interventions. This will ultimately help improve the mental health of college students.

This study identifies exercise adherence as a key mediating variable. Exercise adherence refers to the consistency between an individual’s exercise intention and actual behavior. It explicitly reflects sustained participation in long-term exercise programs (12). As an important predictor of life satisfaction (13), exercise adherence functions through mechanisms explained by Self-Determination Theory. This theory emphasizes three basic psychological needs: autonomy, competence, and relatedness. The satisfaction of these needs directly influences an individual’s level of adherence to exercise. Research indicates that individuals with high exercise adherence tend to maintain consistent physical activity. This ongoing participation fulfills their need for relatedness. They also exhibit increased competence and a stronger social identity. Ultimately, these factors contribute to significantly improved life satisfaction (14). In contrast, individuals with low exercise adherence are often affected by negative psychological factors. These include a tendency towards procrastination and low self-efficacy. Such factors reduce their experience of competence in physical activity settings (15). Moderate exercise adherence promotes mental health by satisfying basic psychological needs. It also establishes a positive exercise–psychology interaction mechanism. This offers a sustainable pathway for enhancing life satisfaction.

Another key mediator in this study is health literacy. Health literacy refers to an individual’s ability to access, understand, and apply health information. It also includes using that information to maintain and improve health (16). As a key factor in health promotion, health literacy is positively correlated with participation in physical activity among adults (17). Studies have shown that the Health Belief Model and the Broaden-and-Build Theory work in tandem. They help explain life satisfaction. Physical exercise improves life satisfaction. Before and after exercise, positive emotions are associated with perceived susceptibility, as predicted by the Health Belief Model. People with high health literacy can detect early signs of mental health issues. They can overcome perceptual barriers. This promotes positive emotions. Ultimately, it improves life satisfaction (18). Recent studies also indicate that health literacy in middle school students is closely linked to participation in school physical activity programs (19). However, existing data indicate that only 25.07% of the Chinese population possesses a high level of health literacy. This shows we need to improve health literacy urgently. Health literacy is a crucial skill for the health and mental development of college students. Improving it can directly raise life satisfaction. It also connects physical exercise and life satisfaction. However, this dual role has not yet been thoroughly studied or proven in current research.

Exercise adherence and health literacy are both closely linked to physical exercise and life satisfaction. They also interact with each other in significant ways. The cognitive theory of emotion helps explain this relationship. When college students feel negative emotions, exercise adherence acts as an important behavioral regulator. It affects how they choose and use coping strategies. In particular, students with high exercise adherence tend to use health knowledge more effectively. They apply it to manage psychological distress and health problems. This creates a positive cycle that strengthens the practical value of health literacy (56). However, several limitations remain in the existing literature. First, the interaction mechanism between exercise adherence and health literacy has yet to be thoroughly investigated. Second, a complete mediating pathway model linking these two variables to life satisfaction has not been established. To address these gaps, an in-depth exploration of the synergistic mechanism between exercise adherence and health literacy is necessary. This will not only enrich the theoretical framework of positive psychology but also provide empirical evidence for developing targeted exercise intervention programs. This holds significant practical value for improving the mental health of college students.

This study proposes the following hypotheses. There are correlations among physical exercise, life satisfaction, exercise adherence, and health literacy (H1). To further investigate the impact of physical exercise on life satisfaction among college students, we developed additional hypotheses. These are based on the Health Belief Model and Self-Determination Theory. We propose that exercise adherence and health literacy mediate the relationship between life satisfaction and college students (H2). Furthermore, university students face challenges due to identity transition. Those with high exercise adherence tend to apply their health knowledge fully. They use effective health techniques to address psychological issues. Therefore, we hypothesize that health literacy and exercise adherence play a mediating role in the chain between physical exercise and life satisfaction (H3).

## 2 Research methodology

### 2.1 Data sources

To improve sample coverage, this study employed cluster random sampling. The sampling frame was based on the 2024 National List of Ordinary Colleges and Universities issued by the Ministry of Education. College students from 37 institutions across four provinces in Central China, namely Hubei, Hunan, Jiangxi, and Henan, were selected as survey participants, reflecting regional variations in life satisfaction. The recruitment period for the study spanned from 08/10/2024 to 09/11/2024, with the subsequent questionnaire survey being conducted from 11/11/2024 to 24/11/2024. The primary reason for focusing on central China is that the college years represent a critical period for identity transformation. Students in this region face considerable academic pressure and exhibit noticeable fluctuations in life satisfaction. The study protocol for this study received approval from the ethics committee at Nantong Health Vocational and Technical College and was documented under approval number 2024(01).

**Figure 1:**
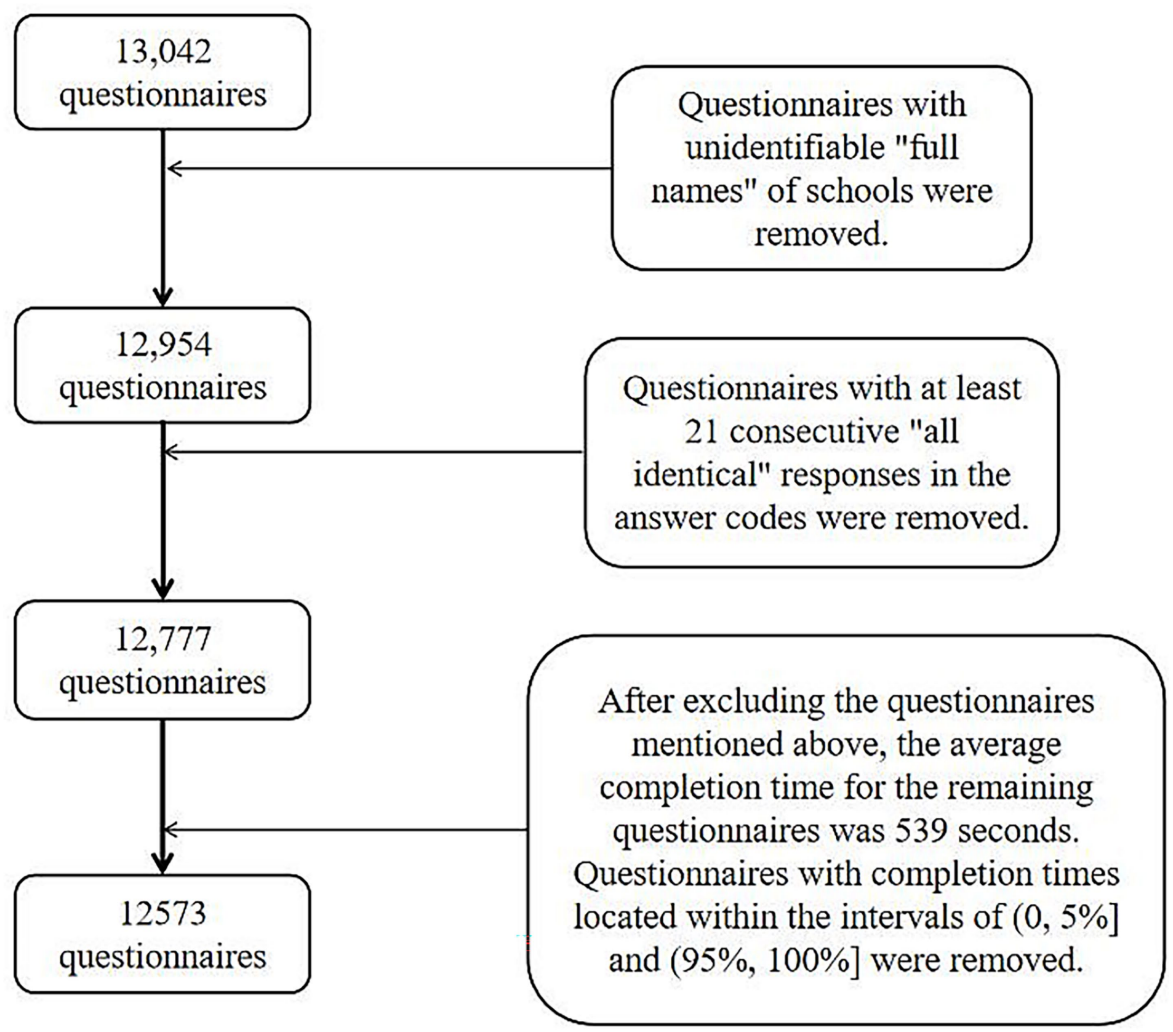
Flow chart of questionnaire screening and sample selection.

Informed consent was obtained from the schools and students. Trained researchers then distributed the questionnaires by class. The responses were collected and screened on site. A total of 13,042 questionnaires were distributed and collected. Invalid questionnaires were excluded. These included patterned responses or failure to follow instructions. After exclusion, 12,573 valid cases were included in the final analysis. Detailed information is presented in Table 1. The data screening process is detailed in Figure 1.

**Table 1:** Sample characteristics table.

Variable	Options	Frequency	Percentage
Gender	Male	5,019	39.9
	Female	7,554	60.1
Grade in school	First grade	8,695	69.2
	Second grade	3,488	27.7
	Third grade	314	2.5
	Fourth grade	76	0.6
Master the skills of movement	0 item	1,374	10.9
	1 item	4,296	34.2
	More than 2 items	6,903	54.9

### 2.2 Measuring tools

(1) Physical activity rating scale (PARS-3)

This study used the physical activity rating scale developed by Japanese scholar Kimio Hashimoto and revised by Liang et al. to measure college students’ physical activity (20, 21). The PARS-3 scale assesses physical exercise amount based on three aspects: intensity, frequency, and duration. It measures the level of participation in physical exercise. In the questionnaire, each item is divided into five levels and scored from 1 to 5. A score of “1″ indicates never participating in physical exercise, while “5″ indicates regular participation. Higher scores reflect greater amounts of physical exercise. The results reflect, to some extent, the physical participation behavior of college students within a specific period. The calculation formula for the amount of exercise is shown in Equation 1:


PAscore=intensity×(time−1)×frequency (1)

According to the normative rating scale for Chinese adults on the PARS-3, scores of ≤19 points indicated small exercise, 20 ~ 42 points indicated medium exercise, and ≥43 points indicated extensive exercise. The test–retest reliability of the PARS-3 is 0.820, and its applicability among Chinese university students has been verified in multiple studies (22–28).

(2) Satisfaction with life scale (SWLS)

The life satisfaction of college students was measured using the SWLS. This scale was developed by American scholar Ed Diener et al. in the 1980s (29). It consists of 5 items, each rated on a 7-point Likert scale. Responses range from 1, indicating strong disagreement, to 7, indicating strong agreement. The total score is the sum of all items, ranging from 5 to 35. Higher scores indicate greater life satisfaction. Chinese scholars Xiong Chengqing and colleagues examined the reliability of the Satisfaction with Life Scale. They used SPSS 13.0 to conduct Cronbach’s alpha and split-half reliability analyses. The results showed the scale’s alpha coefficient was 0.78 and its split-half reliability was 0.70. Both coefficients were relatively high. This indicates the scale has good reliability when measuring life satisfaction in the general population. Therefore, it is considered a valid and reliable instrument for the general population (30).

(3) Exercise adherence

Exercise Adherence as Mediator Variable 1. The Physical Exercise Adherence Scale, developed by Gu et al. from Fujian Normal University, was used to measure college students’ exercise adherence (31). This scale comprises three dimensions: exercise behavior, effort engagement, and emotional experience. It contains a total of 14 items, each rated on a 5-point scale. Higher scores indicate greater individual adherence to exercise. The reliability of the total scale was 0.947, indicating good reliability and validity, making it suitable for assessing physical exercise adherence among college students (31).

(4) Health literacy

Health Literacy as Mediator Variable 2. This study utilized the 9-item Short Form of the Health Literacy Scale (HLS-SF9), adapted from the 12-item Short Form of the Health Literacy Scale (HLS-SF12). The original HLS-SF12 was developed by Tuyen et al. for use in Asian countries (21). Sun et al. later simplified it to form the HLS-SF9 (32). The selection of HLS-SF9 was based on the need for accuracy. The scale consists of three dimensions: health care, disease prevention, and health promotion. Items are rated using four options: “very difficult,” “difficult,” “easy,” and “very simple.” The agreement level (ICC) between HLS-SF9 and HLS-SF12 was 0.989 (95% CI: 0.988–0.999) (33). The HLS-SF9 showed no ceiling or floor effects. Its Cronbach’s alpha coefficient was 0.913, and the split-half reliability was 0.871. The confirmatory factor analysis results for the HLS-SF9 were as follows: χ2/df = 10.844, Goodness of Fit Index (GFI) = 0.985, Adjusted Goodness of Fit Index (AGFI) = 0.971, Normed Fit Index (NFI) = 0.986, Comparative Fit Index (CFI) = 0.987, and Root Mean Square Error of Approximation (RMSEA) = 0.051 (34).

### 2.3 Statistical processing

**Figure 2:**
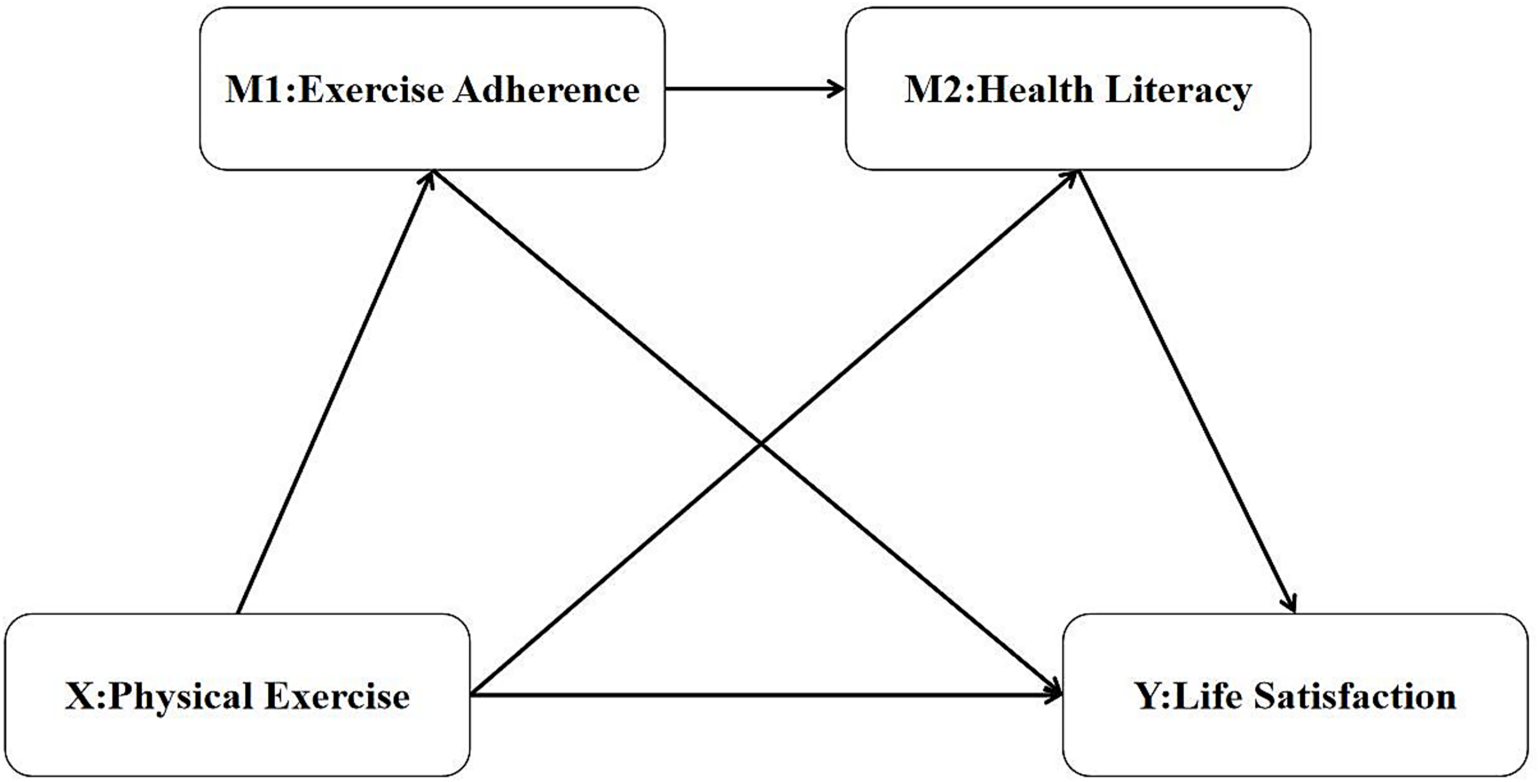
Hypothetical model diagram.

The data processing for this study was conducted using SPSS 27.0 and Excel for all statistical analyses. The process was divided into the following steps:

1. Excel was used to preprocess the data collected from Questionnaire Star. Missing or problematic data points were either retested or removed.
2. This study collected data using self-reported subjective scales. To check for common method bias, a Harman’s single-factor test was performed after data collection. All items related to physical exercise, life satisfaction, health literacy, and exercise adherence were included in an exploratory factor analysis. The results extracted six principal components with eigenvalues greater than 1. The largest factor accounted for 39.7% of the total variance. This value is below the commonly accepted threshold of 40%. Therefore, common method bias was not a concern in this study.
3. Chi-square tests were used to analyze differences in physical exercise and life satisfaction based on gender, grade level, and whether students had mastered a sports skill. Cramer’s V coefficient was used to assess the strength of association between these categorical variables. Its value ranges from 0 to 1, with larger values indicating a stronger correlation. A Cramer’s V greater than 0.1 indicates a weak correlation. A value greater than 0.3 suggests a moderate correlation. A value greater than 0.5 indicates a high correlation (35). Analysis of variance (ANOVA) was used to examine differences in health literacy and exercise adherence. Effect sizes were measured using η^2^, which ranges from 0 to 1. According to Cohen’s criteria, 0.01 represents a small effect, 0.06 represents a medium effect, and 0.14 represents a large effect (35).
4. Pearson correlation analysis was conducted to examine the relationships between physical exercise, life satisfaction, health literacy, and exercise adherence among college students.
5. Regression analysis was used to test for mediation effects. This was further examined using multiple regression analysis via the Process macro. The Bootstrap method was employed for the mediation effect analysis. The proposed hypothetical path model is illustrated in Figure 2.

## 3 Research results

### 3.1 Descriptive analysis

Differences analysis was conducted to assess the physical activity levels among college students with distinct characteristics, as shown in Table 2. The results showed that most college students engaged in a low amount of physical exercise, accounting for 73.1% of the sample. Significant differences were observed by gender (*V* = 0.371), grade (*V* = 0.103), and number of proficient motor skills (*V* = 0.201). Specifically, the proportion of females with low physical activity (86.2%) was significantly higher than that of males (53.5%). Among students proficient in zero motor skills, the proportion of those who engaged in low exercise was highest, at 92.9%. From freshman to senior year, small amounts of exercise consistently accounted for the most significant proportion across grades. In terms of health literacy, the total average score was 27.758 ± 4.086. Significant differences were found by gender (*V* = 0.152), grade (*V* = 0.072), and number of proficient motor skills (*V* = 0.130). Males had higher health literacy scores (28.227 ± 4.520) than females (27.446 ± 3.738). First-year students had the highest health literacy scores among all grades (27.937 ± 4.020), which were significantly higher than those of the other three grades (*p* < 0.001).

**Table 2:** Summary of descriptive analysis of core variables.

Variable	Physical exercise							Health literacy			Life satisfaction			Exercise adherence		
	Low	%	Medium	%	High	%	Statistical value	*M*	*SD*	Statistical value	*M*	*SD*	Statistical value	*M*	*SD*	Statistical value
Total	9,194	73.1	1849	14.7	1,530	12.2		27.758	4.086		22.650	5.556		50.501	8.953	
Gender																
Male	2,685	53.5	1,143	22.8	1,191	23.7	*x*^2^ = 1727.326	28.227	4.520	*x^2^* = 288.654	22.7.1	5.922	*x*^2^ = 155.029	52.848	9.715	*x^2^ =* 875.679
							*p* < 0.001			*p* < 0.001			*p* < 0.001			*p* < 0.001
Female	6,509	86.2	706	9.3	339	4.5	*V* = 0.371	27.446	3.738	*V* = 0.152	22.616	5.299	*V* = 0.111	48.949	8.042	*V* = 0.264
Grade																
First grade	6,380	73.4	1,314	15.1	1,001	11.5	*x^2^* = 267.442	27.937	4.020	*x^2^* = 196.143	22.592	5.507	*x^2^* = 234.424	50.646	8.725	*x^2^* = 437.325
Second grade	2,641	75.7	463	13.3	384	11	*p* < 0.001	27.558	4.216	*p* < 0.001	22.691	5.624	*p* < 0.001	49.791	9.327	*p* < 0.001
Third grade	145	46.2	56	17.8	113	36	*V* = 0.103	27.857	4.348	*V* = 0.072	23.398	5.752	*V* = 0.079	53.063	10.008	*V* = 0.108
Fourth grade	28	36.8	16	21.1	32	42.1		27.500	4.219		24.316	6.709		56.605	7.723	
Mastery of motor skills																
0	1,276	92.9	64	4.7	34	2.5	*x^2^* = 1013.043	26.667	4.054	*x^2^* = 423.338	21.303	5.551	*x^2^* = 282.864	45.502	8.571	*x^2^* = 1219.900
1	3,631	84.5	431	10	234	5.4	*p <* 0.001	27.249	3.824	*p* < 0.001	22.180	5.302	*p* < 0.001	48.942	8.141	*p* < 0.001
More than 2	4,287	62.1	1,354	19.6	1,262	18.3	*V* = 0.201	28.292	4.167	*V* = 0.130	23.211	5.639	*V* = 0.106	52.475	8.950	*V* = 0.220

Health literacy scores increased with the number of proficient motor skills. The highest score (28.292 ± 4.167) was observed among those proficient in more than two skills. For exercise adherence, the total average score was 50.501 ± 8.953. Significant differences were identified by gender (*V* = 0.264) and motor skill proficiency (*V* = 0.220), although the effect size for grade was small (*V* = 0.108). Males scored significantly higher (52.848 ± 9.715) than females (48.949 ± 8.042). Exercise persistence scores were similar across three grades but showed a sharp decline among sophomores (49.791 ± 9.327) (*p* < 0.001). Scores increased with the number of proficient motor skills. Those proficient in two or more skills scored highest (52.475 ± 8.950). Regarding life satisfaction, the overall average score was 22.650 ± 5.556. No significant differences were found by gender (*V* = 0.111), but significant differences existed by grade (*V* = 0.079) and motor skill proficiency (*V* = 0.106). Life satisfaction scores increased gradually with grade level, from 22.592 for freshmen to 24.316 for seniors. Scores also rose with an increase in the number of proficient motor skills.

### 3.2 Correlation analysis

As shown in Table 3, physical exercise is significantly positively correlated with life satisfaction (*r* = 0.137). Life satisfaction also shows significant positive correlations with exercise adherence and its sub-dimensions (*r* = 0.354 to 0.394). Physical exercise is significantly positively correlated with exercise adherence and its sub-dimensions (*r* = 0.363 to 0.486). Health literacy and its sub-dimensions are significantly positively correlated with physical exercise (*r* = 0.149 to 0.219). Health literacy and its sub-dimensions show significant positive correlations with life satisfaction (*r* = 0.302 to 0.362). Finally, exercise adherence and its sub-dimensions are significantly positively correlated with health literacy and its sub-dimensions (*r* = 0.318 to 0.954).

**Table 3:** Overview of correlation analysis results.

Variant	A	B	C	C_1	C_2	C_3	D	D_1	D_2	D_3
A_Physical exercise	1									
B_Life satisfaction	0.137**	1								
C_Exercise adherence	0.458**	0.394**	1							
C_1Exercise behavior	0.486**	0.360**	0.883**	1						
C_2 Effort investment	0.417**	0.371**	0.954**	0.776**	1					
C_3Emotional experience	0.363**	0.354**	0.913**	0.668**	0.836**	1				
D_Health literacy	0.199**	0.362**	0.468**	0.415**	0.441**	0.433**	1			
D_1Healthcare	0.149**	0.302**	0.370**	0.318**	0.347**	0.353**	0.886**	1		
D_2Disease prevention and control	0.168**	0.339**	0.406**	0.376**	0.385**	0.358**	0.916**	0.720**	1	
D_3Health promotion	0.219**	0.333**	0.485**	0.421**	0.456**	0.457**	0.887**	0.668**	0.726**	1

The correlation is significant at the 0.01 level (two-tailed).

### 3.3 Regression analysis

In this study, physical exercise was the independent variable, health literacy and exercise adherence were the mediators,. Gender, grade, and number of motor skills were included as control variables. Life satisfaction was taken as the dependent variable to establish a model testing main, direct, and indirect effects. The results of the hierarchical regression analysis for the chain mediation effect are shown in Table 4. After controlling for gender, grade, and number of proficient motor skills, physical exercise significantly and negatively predicted life satisfaction (*β* = −0.010, SE = 0.003, *t* = −3.596, *p* < 0.001), confirming a primary effect. Furthermore, physical exercise significantly positively predicted exercise adherence (*β* = 0.179, SE = 0.004, *t* = 46.096, *p* < 0.001). It also negatively predicted health literacy (*β* = −0.005, SE = 0.002, *t* = −2.440, *p* < 0.01). Exercise adherence significantly positively predicted life satisfaction (*β* = 0.194, SE = 0.006, *t* = 31.195, *p* < 0.001). Health literacy also significantly predicted life satisfaction (*β* = 0.308, SE = 0.012, *t* = 25.142, *p* < 0.001).

**Table 4:** Hierarchical regression analysis of chain mediation effects.

Regression equation		Overall fit index			The significance of the regression coefficient		
Result variable	Predictor	*R*	*R^2^*	*F*	*β*	*SE*	*t*
Health literacy		0.470	0.221	714.402***			
	Gender				0.031	0.071	0.445***
	Grade				−0.209	0.058	−3.614
	Mastering motor skills				0.189	0.050	3.770***
	Physical exercise				−0.005	0.002	−2.440**
	Exercise adherence				0.215	0.004	52.498***
Exercise adherence		0.482	0.232	950.623***			
	Gender				−0.856	0.154	−5.575***
	Grade				−0.480	0.126	−3.811***
	Mastering motor skills				1.919	0.107	17.857***
	Physical exercise				0.179	0.004	46.096***
Life satisfaction		0.451	0.204	535.492***			
	Gender				0.800	0.097	8.226***
	Grade				0.277	0.080	3.483***
	Mastering motor skills				0.172	0.069	2.502*
	Physical exercise				−0.010	0.003	−3.596***
	Exercise adherence				0.194	0.006	31.195***
	Health literacy				0.308	0.012	25.142***

*** represents *p* < 0.001, ** represents *p* < 0.01, and * represents *p* < 0.05.

### 3.4 Mediating effect analysis

This study employed the bias-corrected percentile Bootstrap method, drawing 5,000 bootstrap samples, using the SPSS PROCESS 4.0 model developed by Hayes (2013). The analysis controlled for gender, grade, and number of proficient motor skills. A chain mediation analysis was conducted to examine the mediating roles of exercise adherence and health literacy in the relationship between physical exercise and life satisfaction among college students. The results of the Bootstrap mediation analysis are presented in Table 5. The main effect of physical exercise on life satisfaction was 0.038, with a confidence interval of [0.033, 0.044]. The total effect was established, as the interval did not include zero. The total indirect effect was 0.052, with a confidence interval of [0.049, 0.056], which does not include zero. The direct effect was −0.014, with a confidence interval of [−0.019, −0.009], also excluding zero. Specifically, the indirect effect through exercise adherence was 0.040 (95% CI [0.036, 0.043]). The indirect effect through health literacy was −0.001 (95% CI [−0.003, 0.000]). The indirect effect through the sequential mediation of physical exercise, exercise adherence, and health literacy on life satisfaction was 0.014 (95% CI [0.012, 0.015]). These results indicate that the model partially supports a chain mediation mechanism.

**Table 5:** Chain mediating effect analysis of college students’ life satisfaction based on the Bootstrap method.

Effect	Effect size	BootSE	95%CI lower limit	95%CI above limit
Total effect	0.038	0.003	0.033	0.044
Total direct effects	−0.014	0.003	−0.019	−0.009
Total indirect effects	0.052	0.002	0.049	0.056
Physical exercise → exercise adherence → life satisfaction	0.040	0.002	0.036	0.043
Physical exercise → health literacy → life satisfaction	−0.001	0.006	−0.003	0.000
Physical exercise → exercise adherence → health literacy → life satisfaction	0.014	0.001	0.012	0.015

**Figure 3:**
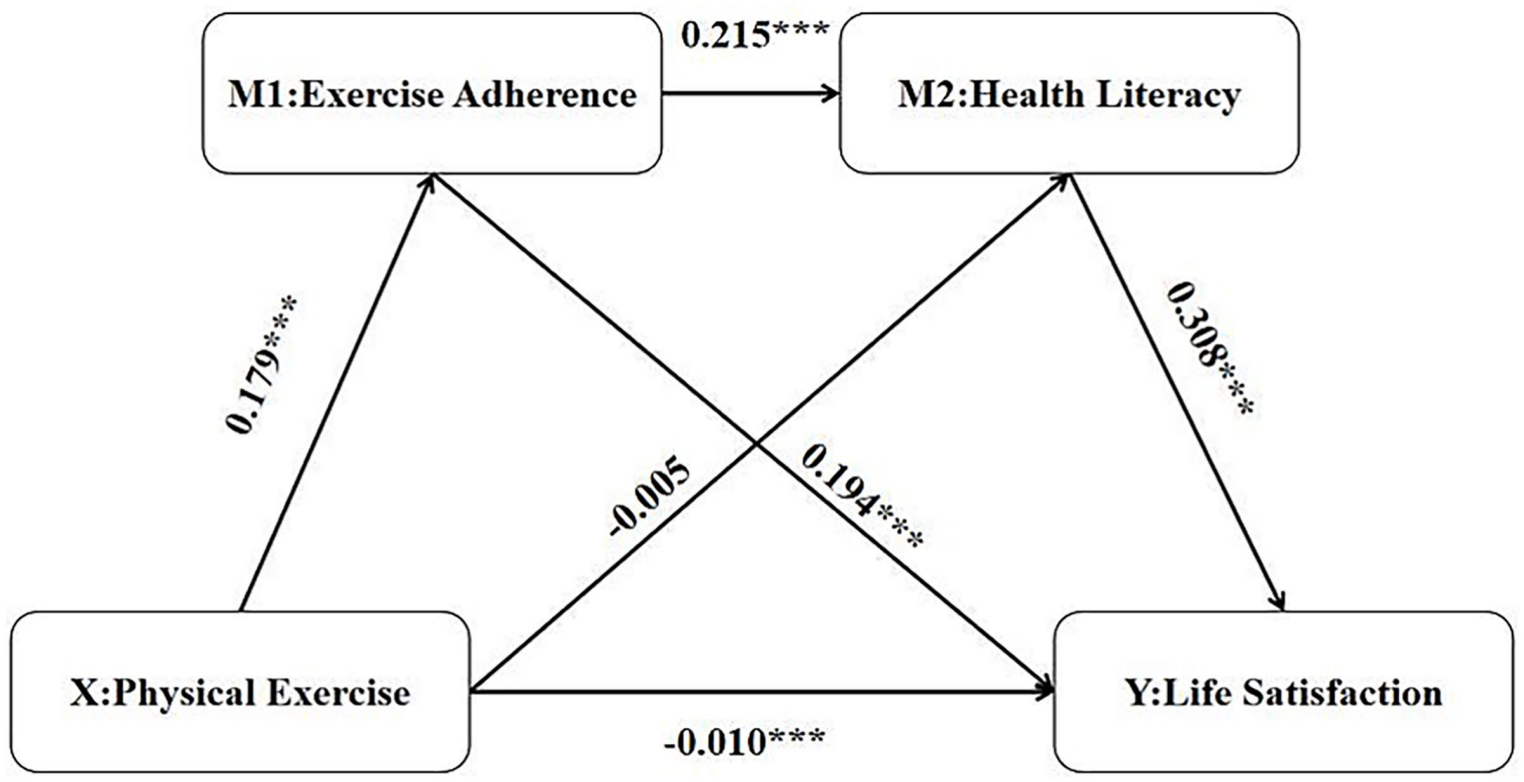
Diagram of the multiple mediation model. *** in the text represents *p* < 0.001, ** represents *p* < 0.01.

The specific values of the total effect, total direct effect, and total indirect effect indicate that exercise adherence, health literacy, and physical exercise each exert a direct influence. These factors collectively affect the life satisfaction of college students. The corresponding effect sizes for each specific pathway are presented in Figure 3.

## 4 Discussion

This study focused on the mechanism through which physical exercise influences college students’ life satisfaction. A chain mediation model was constructed and tested by introducing health literacy and exercise adherence as mediating variables. Health literacy and exercise adherence play key mediating roles in changes in life satisfaction. This highlights the importance of college students’ ability to screen and evaluate their own health status, as well as their capacity for self-determination and autonomy. Considering the relationships among physical exercise, health literacy, exercise adherence, and life satisfaction can provide a scientific basis for designing interventions that comprehensively promote the mental health of college students.

### 4.1 Correlation between physical exercise, life satisfaction, health literacy, and exercise adherence among college students

The results of this study found a weak positive correlation between physical exercise and college students’ life satisfaction. A positive correlation was also observed between physical exercise and health literacy. Furthermore, a moderate positive correlation was identified between physical exercise and exercise adherence. After including mediating variables, physical exercise remained a significant predictor of life satisfaction, supporting hypothesis H1. Identity change plays an important role in the campus life of college students. Good exercise habits provide individuals with a healthy body and richer interpersonal opportunities. They also contribute to higher subjective well-being. Physical exercise is essential for maintaining standard physiological mechanisms and social activities among college students. A lack of exercise may negatively impact their life and academic performance. A cross-sectional study revealed that exercise duration, intensity, and frequency all have varying influences on mental health.

Gender is a significant factor influencing physical performance. Exercise quality in both males and females is related to BMI. This finding differs from the results of this study (36, 37). Other studies have found that mental health is linked to neuroplasticity. Higher physical exercise frequency promotes the growth of neurotrophic factors, thereby significantly improving mental health. Improving exercise adherence enhances life satisfaction through both intrinsic and extrinsic motivation, supporting college students’ pursuit of mental well-being (38). Internationally, self-determination theory suggests that health literacy contributes to life satisfaction by facilitating effective health management. College students with high health literacy and motivation can better understand and apply health skills. This allows them to experience long-term satisfaction from exercise, maintaining motivation and enthusiasm. The rational use of health knowledge helps students view physical exercise as a positive factor in achieving life satisfaction (39, 40). This study also showed a moderate positive correlation between exercise adherence and health literacy. Some researchers have employed various health management plans to enhance the quality of life for patients with intermittent claudication. Questionnaire results after 1 week of exercise showed significant differences in quality of life between experimental groups. Similarly, health management strategies for college students could combine methods such as health literacy training and feedback to maximize effectiveness (41). Previous studies have often focused on exercise adherence in older adults and postoperative populations. As a psychological factor influencing life satisfaction, clear and challenging goals can enhance exercise adherence. Individuals with high exercise adherence are more likely to improve self-achievement and responsibility, thereby reaching specific goals effectively (42).

### 4.2 The direct effect of physical exercise on college students’ life satisfaction

The results of this study indicate that physical exercise does not have a direct, significant positive effect on college students’ life satisfaction. This finding does not directly align with previous research conclusions on the impact of physical exercise on the mental health of different populations. According to the inverted-U dose–response theory, the relationship between physical exercise and life satisfaction is not a simple positive correlation. When the exercise dose falls within an individual’s adaptive threshold range, it can enhance self-efficacy, mood, and self-confidence, thereby improving life satisfaction. However, once the exercise dose exceeds this adaptive threshold, it may lead to chronic stress and increased nervous system load, subsequently reducing life satisfaction (43). For college students in particular, excessively high exercise frequency in the short term fails to generate immediate positive adaptive feedback. Instead, it may result in decreased life satisfaction, ultimately manifesting as a significant negative correlation between the two.

Although physical exercise does not directly and positively influence college students’ life satisfaction, it may still exert a positive impact through other indirect pathways. Self-efficacy and social identity are important driving factors for college students’ adherence to physical activity, and the acquisition of a high social identity may indirectly enhance college students’ life satisfaction (44, 45). According to Rosenberg’s theory of self-esteem, individuals who participate in physical activities, achieve exercise goals, and complete self-challenges can experience a sense of psychological satisfaction and receive positive feedback, thereby enhancing their perceived self-worth (46). Zhou’s research also confirmed that physical exercise can indirectly improve life satisfaction among college students by improving self-control and reducing psychological distress (9). Additionally, the lifestyle and sources of stress among college students may differ from those of other populations, and these factors may partially obscure the direct impact of physical activity on life satisfaction.

### 4.3 The mediating role of health literacy in physical activity and life satisfaction

This study found that health literacy did not significantly mediate the relationship between physical activity and life satisfaction. This suggests that while physical activity influences psychological development and health information management, the effect is not substantial. On the one hand, the direct impact of physical exercise on life satisfaction may be too strong. This could make the mediating role of health literacy less noticeable. Existing studies support the notion that improving health information management enhances health behaviors and individual quality of life, ultimately leading to increased life satisfaction (47).

Additionally, high self-efficacy can help overcome challenges in learning health knowledge. Outcome expectation refers to an individual’s belief in the benefits of healthy exercise. Positive expectations can strengthen health literacy abilities. Together, these factors help improve individual health literacy and quality of life (48).

On the other hand, studies indicate that individual differences in exercise levels and cognitive abilities play an important role. Some college students maintain high health literacy even with low physical activity. Others may fail to acquire health knowledge due to various external factors. These differences may lead to an insignificant mediating effect in the overall sample (49).

### 4.4 The chain mediating role of college students’ physical exercise, life satisfaction, health literacy, and exercise adherence

The results of the mediating effect test showed that the indirect effect of the model was 0.052, which is greater than the total effect of 0.038. This indicates that the mediating effect played a significant role. The chain mediating effect of health literacy and exercise adherence was significantly greater than the direct effect. These results suggest that health literacy and exercise adherence are the main mechanisms through which physical exercise affects life satisfaction among college students. This study proposes a serial mediation model. It examines the indirect pathway from physical exercise to life satisfaction. The pathway operates sequentially through exercise adherence and health literacy. It highlights the crucial role of physical exercise in maintaining mental health. It also reveals potential methods for achieving exercise optimization and positive psychological development through health management.

This study shows that health literacy and exercise adherence serve as key bridges between physical activity and life satisfaction. Adequate physical exercise is a crucial foundation for maintaining healthy management abilities. Rich exercise experience helps supplement health knowledge promptly, promotes the application of health skills, and enhances health literacy (50). Conversely, lack of physical exercise may lead to a decline in health management skills. Based on self-determination theory, when college students engage in activities out of intrinsic interest, satisfaction, or goal achievement, they are more likely to activate both extrinsic and intrinsic motivation. This, in turn, significantly influences goal achievement and enhances individual initiative (57). Good exercise adherence can strengthen the link between self-confidence and stress coping, resulting in a positive persistence effect (51). Moderate exercise adherence is more likely to induce psychological changes in students. It enables positive social interaction among students, providing mutual support and motivation, thereby improving exercise outcomes (42). Using exercise adherence as a mediating variable, this study explored its impact on life satisfaction. The results showed that exercise adherence has a positive influence on life satisfaction. This aligns with findings from scholars such as Ensela Mema. The physiological changes from sustained exercise often improve peer support and self-efficacy. These further promote psychological satisfaction, emotional intimacy, and stability.

Furthermore, persistence in daily physical activity is rooted in self-efficacy. Students’ skills become more proficient after exercise, regardless of their initial preparation. This increase in proficiency not only enhances health literacy but also indirectly contributes to higher life satisfaction.

According to social cognitive theory, improving health literacy depends on individual and environmental influences. Changes in social cognition rely on self-regulatory control and reflective learning of behavioral patterns (52). A longitudinal study provided 90-min weekly health education sessions over 24 weeks to seniors aged 65 and above. The results showed improved gait speed and physical activity levels. It was concluded that exercise instruction and health education lead to physical improvements (53). Some suggest that when exercise intensity approaches the ventilatory or lactate threshold, memory loss or even forgetting may occur. Health literacy may decline due to high oxygen consumption near the end of exercise, often influenced by individual feedback. This finding differs from the results of this study (54). Other studies indicate that health literacy may differ between continuous and interval exercise in adolescents. Slight increases in health beliefs and motor skills were observed before and after exercise throughout the program. This is consistent with the results of this study (55).

Furthermore, a close relationship exists between subjective exercise experience, exercise commitment, and exercise behavior. Moderate exercise adherence has a significant impact on the health literacy of college students. Ample exercise opportunities help maintain high health literacy. Health management ability influences goal setting and exercise commitment, promoting positive overall exercise behavior and mental health by improving health literacy.

### 4.5 Research limitations

This study finds that physical exercise has a significant negative correlation with college students’ life satisfaction, indicating a direct negative effect. However, it may also indirectly contribute to life satisfaction by enhancing exercise adherence and health literacy. These findings suggest that when implementing integrated physical activity and health strategies to promote student well-being, it is crucial to consider these complex mechanisms.

One limitation of this study is the use of self-reported data. Self-reports can capture participants’ subjective feelings and perceptions. They also allow results to be communicated quickly and easily. However, the reported data may be subject to response bias. The results can also be influenced by individuals’ cognitive levels and emotional states. This study sampled Chinese university students, and its findings may be limited in their generalizability to student populations in other countries.

## 5 Conclusion

This study aimed to investigate the significant role of health literacy and exercise adherence in the life satisfaction of college students. Physical exercise shows a strong correlation with college students’ life satisfaction. Health literacy and exercise adherence together form a complete chain mediating mechanism in this relationship. By exploring the chain mediation of exercise adherence and health literacy and analyzing the internal connections among these factors, this study helps to broaden the understanding of how life satisfaction is influenced among college students. These findings have positive social implications for promoting healthy exercise habits and enhancing the physical and mental health of college students. They also offer a practical foundation for designing more comprehensive intervention programs aimed at improving life satisfaction in this population.

## Data Availability

The data analyzed in this study is subject to the following licenses/restrictions: data cannot be shared publicly, because data from this study may contain potentially or sensitive patient information. Data are anonymized, however nevertheless, due to relatively few severe cases, patients could be identified. Therefore, data from this study will be made available for researchers who meet criteria for access to confidential data. The datasets used and/or analyzed during the current study available from the corresponding author on reasonable request. Requests to access these datasets should be directed to Rong-hai Luo LRH_066@163.com.
